# CPIRD: A successful Thai programme to produce clinically competent medical graduates

**DOI:** 10.12688/f1000research.6638.1

**Published:** 2015-06-18

**Authors:** Yanhua Yi, Virasakdi Chongsuvivatwong, Hutcha Sriplung, Chulalak Rueanarong

**Affiliations:** 1 Epidemiology Unit, Faculty of Medicine, Prince of Songkla University, Hat Yai, Songkla, 90110, Thailand; 2 Faculty of Medicine, Prince of Songkla University, Hat Yai, Songkla, 90110, Thailand

**Keywords:** rural, grade point average (GPA), license examination, clinical competency, CPIRD

## Abstract

The programme titled “Collaborative Project to Increase Production of Rural Doctors” (CPIRD) is a rural medical education project launched in 1994 in Thailand. This study aimed to compare the academic performances in medical study over five years and the pass rates in national medical license examinations (MLE) between students enrolled in CPIRD and two other tracks.

Grade point average (GPA) over five years and results of MLEs for four cohorts of students enrolled from 2003 to 2006 in Prince of Songkla University were collected from the registration department. A longitudinal analysis was used to compare the GPA over time for medical students enrolled in CPIRD and those from the national and direct regional tracks through generalized estimating equation (GEE) models. The MLE pass rates were compared using chi-square and fisher's exact tests as appropriate.

Female students dominated the CPIRD group. GPAs in the first three years in the CPIRD group were significantly lower than those of the other two groups, this disparity narrowed in the fourth and fifth years. For step one of the MLE (basic sciences), cohorts 2003 and 2006 of the CPIRD group had a significantly lower pass rate than the other two groups but there was no significant difference in cohort 2004 and cohort 2005. The CPIRD step two and three MLE pass rates were not significantly different from the national track in all cohorts and lower than the direct track only for step two in cohort 2003 and step three in cohort 2006. The step three pass rate of the CPIRD group in cohort 2004 was significantly higher than the other two tracks.

Despite weaker competency in basic science, the CPIRD was successful in forming clinical competency.

## Introduction

In 2010, the World Health Organization (WHO) recommended sixteen interventions to improve health force retention in rural areas. These included education strategies to recruit students of rural origin, locating medical schools outside major cities, bringing students to rural communities and matching curricula with rural health needs ^1^.

Thailand is well known for its emphasis on rural health development ^2^. Since 1972, all medical graduates must spend at least three years of compulsory service in rural areas. In the same year, the medical school of Prince of Songkla University (PSU) was established in southern Thailand, the most remote part of the country, in order to strengthen the local capacity in medical services. From the initial establishment period, PSU had two kinds of enrollment methods. The first is a national entrance examination (hereafter abbreviated to national track), which allows students from all over Thailand to sit the examination for a chance to study ^3^. For geographic and socio-cultural reasons, this medical school in the south has not been a popular choice for candidates from high schools in other regions of the country. The local medical school compensates for this by using a second method of recruitment called the direct admission programme (hereafter abbreviated to direct track). This method recruits students from the southern regional provinces exclusively based on an institution-specific examinations ^4^, which take place a few months before the announcement of the national track examinations. This earlier announcement makes the programme popular to local candidates because they get admitted earlier and naturally have no difficulties acclimatizing to the different culture in the south of the country ^4^. For decades, direct track students are known to have a better average academic performance than the national track students ^4– 6^.

To further ensure adequate supply of medical doctors to the rural region, especially the potential insurgent areas of southern Thailand, a third track was introduced in 2003. Under the “Collaborative Project to Increase Production of Rural Doctors” (CPIRD), rural students from the region were recruited with a longer period (six years) of obligatory service in specific areas where there were a shortage of doctors. Later, the “One District One Doctor (ODOD)” programme was brought in as the fourth track ^4^. ODOD students were not included in this analysis as the programme was considered too new.

Students of all tracks complete the first three years of medical study together. The national track and direct track students take their following three years of clinical study in university hospitals and CPIRD students in accredited regional and provincial hospitals of the Ministry of Public Health (MOPH) ^4^. Grade Point Average (GPA) was used to assess the student’s performance in all years and was based on the same standard set assessed by the regional medical university.

Since 2002, the Thai Medical Council has required all medical students who matriculated from the year of 2003 to pass all three parts of the Medical Licensing Examination (MLE) before getting their medical licenses ^7^. The three steps of the MLE are taken at the end of the third, the fifth and sixth year, respectively. The first step of the MLE focuses on basic science knowledge, the second step on clinical science knowledge, and the third step on both knowledge and clinical skills evaluation. This is to standardize the basic competencies of graduate physicians and to assure health consumers have a standard health care service ^8^.

While the idea of recruiting medical students from rural areas and training them at hospitals close to the rural population is highly advocated based on the findings that it had positive implication on rural retention ^9– 12^, but competency of graduates from such programmes have rarely been investigated.

The main objective of this study was therefore to compare the academic performance of the students recruited from different tracks as reflected by their GPA over five years and the pass rate at each step of the MLE.

## Methods

### Study site

Southern Thailand, where this study was conducted, is a region of the country with the highest levels of heterogeneity of the population and continuous ethnic unrest ^13^.

### Study design

A retrospective cohort study based on the records of the performance of all medical students enrolled in 2003 to 2006 was used.

### Dataset and ethical clearance

The data was retrieved from the student registry of Faculty of Medicine, PSU. All personal identification was encrypted. The study protocol was approved by the Ethics Committee of the Faculty of Medicine, PSU (Permit No: 56-317-18-5).

### Data analysis for academic performance of GPAs and MLE results

All data analyses were performed using R version 3.1.3 ( http://www.r-project.org) and Epicalc package 2.15.1.0 ( http://CRAN.R-project.org/package=epicalc). A longitudinal data analysis was used to compare the GPA over five years for medical students enrolled in three different programmes through generalized estimating equation (GEE) models. The results for the pass rates in MLE were analyzed using chi-square and fisher's exact test as appropriate. Statistical significance was set at 5%.

## Results

Medical students academic performance and MLE results for four cohorts Admission indicates three different tracks including CPIRD, Direct track and National track; NLE indicates National License Examination; Yr1–Yr5 indicates First to Fifth year GPA. Click here for additional data file. Copyright: © 2015 Yi Y et al. 2015 Data associated with the article are available under the terms of the Creative Commons Zero "No rights reserved" data waiver (CC0 1.0 Public domain dedication).

Table 1 compares baseline characteristics of the students from the three tracks. Female students had a larger percentage in the CPIRD group compared with direct track and national track students. The number of students in the CPIRD increased from 19 in 2003 to 72 in 2006, whereas students from other two programmes remained stable.

**Table 1.** Student characteristics by the three enrollment programmes.

Characteristic		CPIRD N=171	Direct Track (N=208)	National Track (N=252)	Total (N=631)
Sex***					
	Female	119 (69.6)	98 (47.1)	148 (58.7)	365(57.8)
	Male	52 (30.4)	110 (52.9)	104 (41.3)	266(42.2)
Year of Enrollment***					
	2003	19 (11.1)	57 (27.4)	48 (19.0)	124(19.7)
	2004	27 (15.8)	48 (23.1)	87 (34.5)	162(25.7)
	2005	53 (31.0)	45 (21.6)	66 (26.2)	164(26.0)
	2006	72 (42.1)	58 (27.9)	51 (20.2)	181(28.6)

Numbers in bracket are percent unless otherwise stated. *** p-value <0.001, ** p-value <0.01, * p-value <0.05

CPIRD: Collaborative Project to Increase Production of Rural Doctors

**Figure 1.**
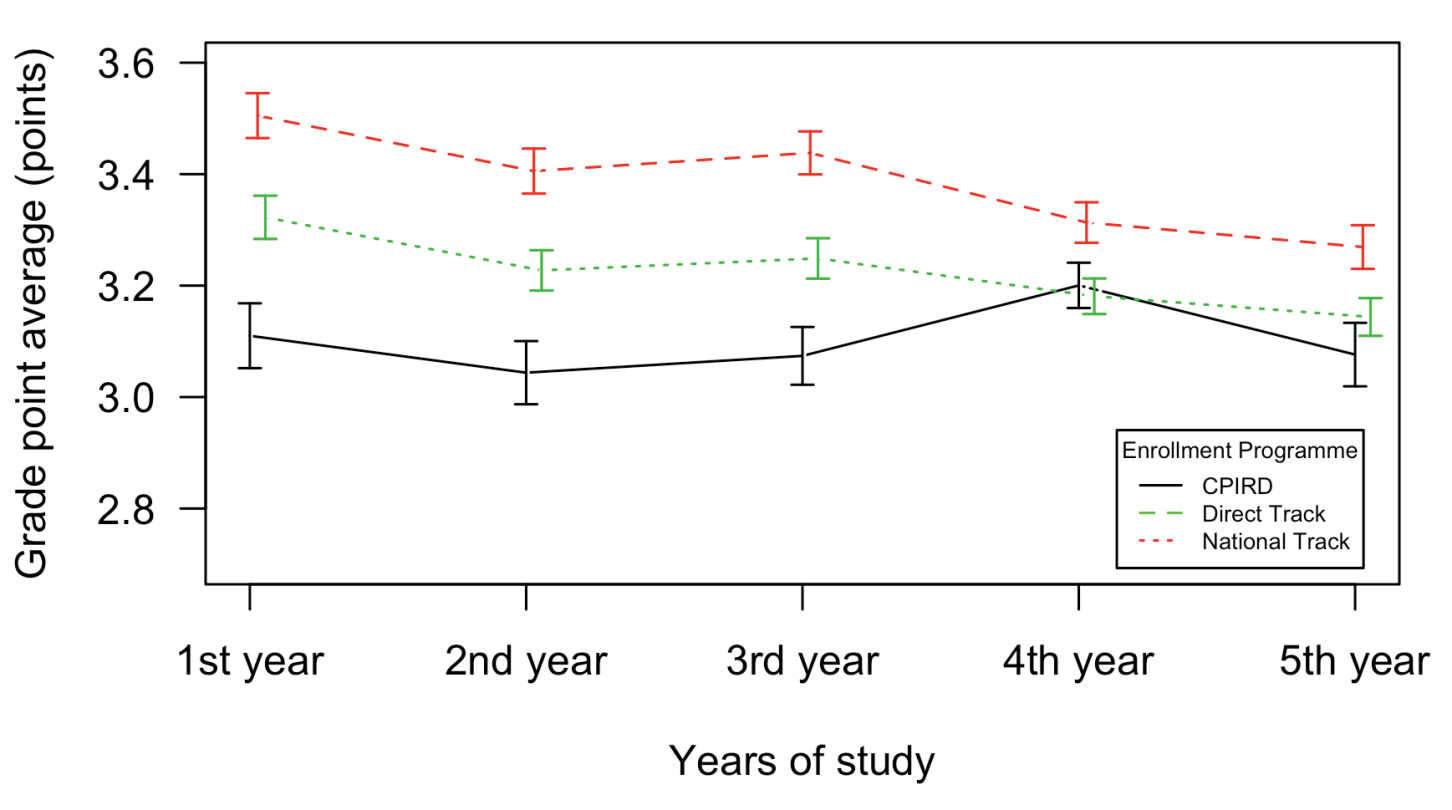
GPA over five years by three different programmes.

Figure 1 shows how the GPA changed over five years of time. In the first three years, the mean GPA of students from three enrollment programmes was significantly different. Students from the direct track performed best. Followed by national track students. Students from CPIRD had lower GPAs than the others. However, the GPAs of the last two years from the three groups tended to converge. Table 2 summarizes results of the GEE with ‘year’ as a continuous variable and ‘track’ as a categorical variable. Their interaction was statistically significant; therefore the interaction terms were included in the final model. Non-significant positive coefficient for the main effect ‘year’ indicates that the tendency of increment of average performance scores of the reference group (CPIRD) was not significant. The other two main effects ‘direct track’ and ‘national track’ were both significantly higher than that of CPIRD in the reference year (first year). Both interaction coefficients are negative indicating that over the years, the difference between the two tracks and CPIRD was reduced significantly.

**Table 2.** Results of the generalized estimating equations analysis on the relationship between GPA and enrollment programmes.

	Coef. ^†^	SE	p-Value
Year of study	0.009	0.009	0.320
Enrollment programmes (ref: CPIRD)			
Direct Track	0.482	0.037	<0.001
National Track	0.272	0.036	<0.001
Interaction terms Year: Direct Track	-0.065	0.011	<0.001
Year: National Track	-0.049	0.010	<0.001

^†^ Coef: Coefficient; SE: Standard error

Table 3 shows the association between enrollment programmes and results of the MLE for the four cohorts of students enrolled from 2003 to 2006. In step one of the MLE, CPIRD students were weaker than students in the other tracks for cohort 2003 and cohort 2006. In the remaining two MLE steps, CPIRD students’ pass rate was not statistically different from that of the national track students. Direct track students had a higher pass rate only in step two of the MLE in cohort 2003 and step three of the MLE in cohort 2006 compared with CPIRD students. In fact, CPIRD had the highest pass rate in step three of the MLE in cohort 2004.

**Table 3.** Association between enrollment programmes and results of medical license examinations stratified by cohorts.

	Step 1			Step 2			Step 3		
	Fail (N)	Pass (N)		Fail (N)	Pass (N)		Fail (N)	Pass (N)	
Cohort 2003			P-value			P-value			P-value
CPIRD	8	11	…	3	16	…	5	14	…
Direct Track	0	57	<0.01	0	57	<0.05	5	52	0.12
National Track	5	43	<0.01	2	46	0.26	9	39	0.72
Cohort 2004									
CPIRD	5	22	…	0	27	…	2	25	…
Direct Track	5	43	0.52	0	48	1	15	33	<0.05
National Track	22	65	0.64	5	82	0.46	31	52	<0.01
Cohort 2005									
CPIRD	19	34	…	3	50	…	2	51	…
Direct Track	8	37	0.08	1	44	0.73	2	43	1
National Track	18	48	0.42	1	65	0.46	10	56	0.08
Cohort 2006									
CPIRD	26	46	…	3	69	…	16	52	…
Direct Track	5	53	<0.01	2	56	1	5	53	<0.05
National Track	7	44	<0.05	2	49	1	7	42	0.31

N: the number of students … indicates referent group

## Discussion

CPIRD students had a lower GPA on average in pre-clinical years and lower pass rates of the MLE in basic science parts than students of the other two tracks. Their GPA tended to catch up with their peers in clinical years and the pass rate of the MLE in the clinical parts were more or less comparable with their peers.

The selection process of medical students in Thailand could explain the fact that CPIRD students had the lowest GPA in the first three years. Direct track students were those students in southern Thailand with good academic records who sat for the entrance examination at Prince of Songkla University. National track students selected Prince of Songkla University as an alternative choice because of its geographic distance from Bangkok. CPIRD students were those unable to get through by direct track examination and finally selected by the CPIRD route. As a result, direct track students had the highest academic performance in high school, followed by national track students, while CPIRD students were weakest ^4^. The first three years was the pre-medical and pre-clinical study. It has been shown in other medical schools that the pre-clinical stage including second and third year, had a high correlation with the first year pre-medical stage ^3^. The lower performance in these first three years for CPIRD students reflected their weaker background and performance in science and thus these students need support to reduce the dropout rate ^14, 15^.

A previous study suggested that CPIRD students had more opportunities to practice in regional hospitals and thus displayed more capable clinical skills in the fourth and fifth year ^3^. In addition, the successful application of problem-based learning (PBL) in clinical study reduced the difference in academic performance and fostered a self-motivated study atmosphere among medical students ^16^.

The findings that the CPIRD students could perform as good as those normal tracks of students in step two and step three MLE has important implications. Good clinical education does not need to be confined to a conventional teaching hospital. Decentralized medical education requires enhancement of existing hospitals. The byproducts of this strengthening include increasing service capacity and quality of health services to local populations, which reduces the inequality problems due to geographical barriers. Other studies also reported that Thai CPIRD doctors were more likely to stay longer in rural areas than their peers ^17, 18^. Most low and middle-income countries (LMICs) have a serious rural–urban disparity of health service and the clinical education is mostly based in university teaching hospitals in large cities ^19^. The experience from the Thai CPIRD should therefore be carefully reviewed for potential adaptation to other low LMICs.

### Limitations

This was a retrospective study; other factors influencing academic performance could not be determined and taken into account. Examination questions and behavioral performance of the students may differ with time and place. However, the MLE were rigorously standardized national examinations and comparisons across student groups were made mainly within the same cohort. Thus, this limitation has been minimized.

## Conclusion

The CPIRD was successful in creating clinically competent doctors despite lower GPAs in the pre-clinical year.

## Data availability

The data referenced by this article are under copyright with the following copyright statement: Copyright: © 2015 Yi Y et al.

Data associated with the article are available under the terms of the Creative Commons Zero "No rights reserved" data waiver (CC0 1.0 Public domain dedication). 

F1000Research: Dataset 1. Medical students academic performance and MLE results for four cohorts, 10.5256/f1000research.6638.d49930 ^20^

## Supplementary material

**Data analysis using R and Epicalc.**

Data analysis and output for Table 1, Figure 1, Table 2 and Table 3.
