# Supplementary material for: CPIRD: A successful Thai programme to produce clinically competent medical graduates
Source: F1000Res. 2015 Jun 18;4:158. [Version 1] doi: 10.12688/f1000research.6638.1 (PMC4648210; doi:10.12688/f1000research.6638.1)
Supplement: Peer review history

# Peer review: Referee response for version 1

**DOI:** 10.5256/f1000research.7131.r9407

**Published:** 2015-07-20

**Authors:** Shama Virani, Katie Rentschler

**Affiliations:** 1 Department of Environmental Health Sciences, University of Michigan, Ann Arbor, MI, USA

CPIRD: A successful Thai programme to produce clinically competent medical graduates

**This research describes differences in GPA and MLE scores of medical students that enter the program through three different mechanisms. These mechanisms seem to have an impact on initial scores, but become comparable at the end of the program.**

Comments:

This paper is integral in future implementation of intervention programs designed to retain health forces in rural areas. Through this analyses, the authors have shown that clinical experience is as valuable as the curriculum.

Minor revisions are suggested below.

Page 1

 

- Is there a rationale as to why step one MLE scores of the CPIRD group was significantly lower for only cohorts 2003 and 2006 but not 2004 and 2005?
- Figure 1
  This figure shows that national track students have the best GPAs overall. It seems to contradict the statements in the introduction and discussion that mention direct track students do better than national track. It would be useful to address this discrepancy in the discussion.

Page 3:

- Is there information on how many students in CPIRD first try to attend medical school through the national or direct tracks?
- For the study site, please include the university where the study took place

Page 4:

- Is there any particular reason why the CPIRD group contains more females? Did this change over time? If so, it might be useful to include gender as a covariate in the model as this might influence the trajectory of the scores.
- Table 3: Please indicate the test used to obtain p-values in the table legend. It might also be useful to bold the “Cohort 2003”..etc to make the table a bit easier to read

Level of Interest:  An article of importance in its field

We have read this submission. We believe that we have an appropriate level of expertise to confirm that it is of an acceptable scientific standard.

# Peer review: Referee response for version 1

**DOI:** 10.5256/f1000research.7131.r9408

**Published:** 2015-07-09

**Authors:** Weerasak Putthasri

**Affiliations:** 1 International Health Policy Programme (IHPP), Ministry of Public Health, Nonthaburi, Thailand

This manuscript generates concrete evidence to support the WHO 2010 recommendation focusing on the graduates' competencies. To disseminate this academic proof is important and useful for both national and global audiences. However, there are some minor points could be improved for paper quality, as follows

1. Table 1, please check 'Percentage' of the national track column. The total percent must be 100.0%.
2. As for the MLE pass rate, the 'Fail' was students who did not pass the exam on 'only the first attempt of that test', correct? Please describe more.
3. Discussion:
  Authors introductory mentioned the WHO global policy recommendations on increasing access to health workers in remote and rural areas through improved retention and want to prove the competencies or performance of that implementation. In order to link this evidence to policy recommendation, authors may consider to add some sentences using this key finding to support the global recommendation implementation, esp. Students from rural backgrounds and Clinical rotations in rural areas during studies which are the key characteristic of CPIRD. Further more, authors could also consider to mention the WHO recommendations on transforming and scaling up health professionals' education and training, 2013 which has recommended for targeted admissions policies to increase the socio-economic, ethnic and geographical diversity of students and the expansion of faculty through recruitment of community-based clinicians and health workers as educators, as well.  

I have read this submission. I believe that I have an appropriate level of expertise to confirm that it is of an acceptable scientific standard.

# Peer review: Referee response for version 1

**DOI:** 10.5256/f1000research.7131.r9104

**Published:** 2015-07-01

**Authors:** Supasit Pannarunothai

**Affiliations:** 1 Centre for Health Equity Monitoring, Faculty of Medicine, Naresuan University, Phitsanulok, Thailand

The paper is concise and clear, providing evidence that medical students from Collaborative Project to Increase Production of Rural Doctors (CPIRD) tract finally watched up with medical students from local direct admission and national admission.

There are some points to improve the quality of this paper:

1. The text explaining the CPIRD programme in paragraph 3 of Introduction is not accurate. The obligatory service period of service is still kept constant at three years not six years as written. The One District One Doctor (ODOD) programme coerces 12 years of obligatory service.
2. Methods section should explain clearer on selection of students especially about drop-out between years. It's unlikely that there were no dropouts during the 6-year MD programme. How the study handle this issue? What bias is likely to occur? Please discuss.
3. The GPA presentation in figure 1 shows that students of the national tract best performed since the first year, but table 2 and text in result section explained that students of direct tract best performed. Please check.
4. Table 3 shows very high fail rate of step 3 exam as compared to others. There is no explanation how the systems interacted with this high fail rate.

I have read this submission. I believe that I have an appropriate level of expertise to confirm that it is of an acceptable scientific standard.
